# The Role of Lymphatic System Dysfunction, IL-33, and Neutrophil Extracellular Traps in Rheumatoid Arthritis: A Comprehensive Review

**DOI:** 10.3390/cimb48101034

**Published:** 2026-10-06

**Authors:** Mi Yang, Li Zhang, Chang-Hao Luo, Bin-Bin Zhang, Jia-Nan Zhao

**Affiliations:** 1 College of Pharmaceutical and Chemical Engineering, Shaanxi A&F Technology University, Yangling 712100, China; 2 School of Clinical Medicine, Hangzhou Normal University, Hangzhou 311121, China; 3 College of Chemical & Pharmacy, Northwest A&F University, Yangling 712100, China; 4 Department of Cardiovascular Sciences, Temple University, Philadelphia, PA 19140, USA

**Keywords:** rheumatoid arthritis, autoimmune disease, neutrophil extracellular traps, lymphatic endothelial cell, IL-33/ST2 axis

## Abstract

Rheumatoid arthritis (RA) is sustained not only by immune activation within the synovium but also by dysfunction of the lymphatic network that normally clears interstitial fluid, inflammatory mediators, and leukocytes migrating from inflamed tissues. Functional abnormalities in lymph node stromal cells may arise before clinically apparent arthritis. After RA becomes established, the lymphatic response may shift from compensatory lymphangiogenesis to reduced collecting-vessel contraction, structural deterioration of draining lymph nodes, and impaired lymphatic drainage. This review examines how IL-33/ST2 signaling, neutrophil extracellular traps (NETs), and the lymphatic endothelial program maintained by PROX1 may influence the shift from compensatory remodeling to lymphatic dysfunction. During early inflammation, IL-33 can activate the ST2/TRAF6/PI3K/Akt/eNOS/NO pathway in lymphatic endothelial cells (LECs), while VEGF-C/VEGFR3 signaling supports lymphatic vessel growth and fluid transport. But vessel expansion does not guarantee that newly formed lymphatics mature or restore effective drainage. As inflammation persists, TNF-driven iNOS activity, occupation of lymphatic sinuses by B cells, and structural changes in draining lymph nodes can reduce lymphatic transport and prolong synovial exposure to cytokines, immune complexes, and autoantigens. NETs directly injure lymphatic endothelium in inflammatory models outside RA, but direct evidence in synovial LECs remains unavailable. NET-associated histones, proteases, myeloperoxidase, extracellular DNA, and HMGB1 should therefore be considered plausible rather than established mediators of LEC injury through junctional disruption, oxidative stress, or RAGE- and TLR9-related signaling. Reduced PROX1 activity may further weaken lymphatic endothelial identity by disrupting the expression of VEGFR3, LYVE1, PDPN, and CCL21, which support vessel maintenance and immune-cell transport. Based on these findings, we propose a stage-dependent model in which established IL-33–NET–fibroblast-like synoviocyte and IL-33–macrophage circuits increase the inflammatory burden, while declining lymphatic clearance retains the mediators that sustain these circuits. This framework separates demonstrated mechanisms from untested links and identifies synovial LEC injury, lymphatic transport, and stage-specific pathway modulation as priorities for mechanistic studies and therapeutic development.

## 1. Introduction

Rheumatoid arthritis (RA) is a systemic autoimmune disease affecting approximately 1% of the global population and occurs about three times more frequently in women than in men [1]. The main clinical features of RA are joint inflammation and structural damage. But lymphatic dysfunction can also play a role in the development and progression of disease. Under physiological conditions, lymphatic vessels drain excess interstitial fluid and transport immune cells from peripheral tissues to lymph nodes (LNs). These processes play an important role in maintaining tissue immune homeostasis and orchestrating adaptive immune responses [2]. In RA, impaired lymphatic drainage may result in retention of pro-inflammatory cytokines, immune cells and tissue debris within the synovium, sustaining inflammation and contributing to joint damage [3]. Lymph node stromal cells (LNSCs) are critical for immune tolerance, and their dysfunction may occur early in RA [4,5,6]. Defective lymphatic flow may also affect peripheral tissues, as suggested by enhanced cutaneous cytokine retention and psoriasis-like inflammation in a mouse model of lymphatic dysfunction [3,7]. A similar mechanism could operate in RA, where decreased lymphatic clearance from the synovium may retain inflammatory mediators that drive synovial hyperplasia and progressive joint destruction. Lymphatic abnormalities may also be intertwined with extraarticular complications of RA, although direct evidence for associations with cardiovascular disease or neuropathy is limited and should be distinguished from the well-established contributions of blood-vessel endothelial dysfunction and impaired microvascular perfusion [1]. Together, these observations provide a rationale for investigating whether restoration of lymphatic drainage may reduce synovial inflammation and improve systemic outcomes in RA.

Interleukin-33 (IL-33), a member of the IL-1 cytokine family, is sequestered in the nucleus of intact cells, but released as an alarmin upon cellular or tissue injury [8,9]. IL-33 has a dual function in immune regulation and inflammation; consequently, its effects on autoimmune disease are context-dependent and influenced by the surrounding cellular and cytokine milieu. IL-33 drives type 2 immunity by activating group 2 innate lymphoid cells (ILC2s), Th2 cells and mast cells. These responses, depending on the tissue context, can be involved in tissue repair and immune regulation [10,11]. IL-33 can also activate effector immune cells and increase production of pro-inflammatory cytokines in autoimmune inflammatory conditions [12,13]. Data from experimental models of arthritis and from patient samples support this context-dependent activity in RA. For example, IL-33-deficient and wild-type mice developed comparable severity of collagen-induced arthritis (CIA), suggesting that endogenous IL-33 was not required for disease development under these experimental conditions [14]. Conversely, administration of exogenous IL-33 inhibited CIA in C57BL/6 mice, which suggests that pharmacologic IL-33 exposure can elicit effects that differ from those of endogenous IL-33 [14]. In RA patients, high serum and synovial fluid IL-33 levels have been noted, which correlate with disease activity [9]. The regulatory role of IL-33 in inflammation is also supported by evidence from other autoimmune diseases. Findings in Sjögren’s syndrome (SS) also demonstrate how the local inflammatory environment modulates IL-33 activity [15]. In SS, dysfunctional salivary gland epithelial cells secrete IL-33, which, in collaboration with IL-12 and IL-23, enhances the production of IFN-γ by natural killer (NK) and natural killer T (NKT) cells, resulting in enhanced glandular inflammation [13]. In other immune settings, IL-33 can activate regulatory T cells (Tregs) to limit excessive immune activation [9]. Collectively, these results suggest that the effects of IL-33 are dictated by the responding cell population, the cytokine milieu, and the time and stage of disease. Therapeutic strategies targeting this pathway will therefore need to differentiate between situations where IL-33 promotes immune regulation versus where it promotes inflammation.

Neutrophil extracellular traps (NETs) are web-like structures that contain decondensed chromatin, histones, and neutrophil-derived antimicrobial proteins and are released in response to microbial or inflammatory stimuli [16]. Although NET formation can support antimicrobial defense, excessive or poorly cleared NETs may promote autoimmune inflammation, including the inflammation associated with RA [17]. In RA, increased NET formation has been detected in synovial fluid and synovial tissue. NET-associated molecules may sustain local inflammation, expose autoreactive lymphocytes to modified antigens, and contribute to cartilage and bone damage [18]. NETs may influence RA inflammation by stimulating immune cells and by carrying proteases, histones, and other molecules that can damage surrounding tissues [19]. IgA-containing immune complexes detected in the plasma and synovial fluid of patients with RA can induce NET formation through FcαRI expressed on neutrophils [18]. The resulting NETs contain neutrophil elastase, myeloperoxidase (MPO), and other granule-derived enzymes. NET-associated proteases can degrade extracellular matrix proteins and may thereby contribute to cartilage and bone erosion [16]. NETs can also expose citrullinated proteins and other modified molecules that may serve as autoantigens recognized by anti-citrullinated protein antibodies (ACPAs) and other autoantibodies in RA [20]. This interaction may establish a positive-feedback loop in which NET-derived autoantigens support autoantibody responses, while immune complexes containing these autoantibodies stimulate further NET formation. NETs may also contribute to the vascular endothelial dysfunction that occurs in RA [21]. In a microvascular injury model outside the context of RA, Boneschansker et al. reported that NET accumulation disrupted capillary perfusion by separating red blood cell movement from plasma flow, which promoted tissue hypoxia and secondary organ injury [22]. A similar process could aggravate synovial inflammation in RA if NET-mediated endothelial injury reduces local perfusion or increases vascular permeability. NET components can also activate complement pathways and stimulate surrounding immune or stromal cells to produce cytokines such as TNF-α and IL-6, thereby reinforcing local inflammation [23]. Together, these findings support further evaluation of approaches that limit excessive NET formation, neutralize NET-associated toxic molecules, or improve NET clearance in RA.

## 2. Stage-Dependent Lymphatic Remodeling in Rheumatoid Arthritis

Lymphatic remodeling in RA does not follow a single pathological pattern, but rather changes as the synovial inflammation progresses. During early inflammatory stages, lymphatic vessel dilation and vascular endothelial growth factor C (VEGF-C)-dependent lymphangiogenesis may promote fluid clearance and immune-cell transport. Chronic inflammation can lead to impaired vessel maturation, decreased collecting-vessel contraction, and impaired lymph node function, resulting in a lymphatic response that shifts from compensation to drainage failure. This section discusses the stage-dependent transition, the interactions between lymphatic vessels and synovial immune cells that control this process, and the methodological limitations that prevent the exact determination of its timing and clinical importance.

### 2.1. Compensatory Lymphangiogenesis During Early-Stage Inflammation

In early RA, lymphatic vessels are remodeled in a compensatory manner to increased interstitial fluid and infiltration of inflammatory cells into the synovium [6,24]. This process is characterized by both the dilation of existing lymphatic vessels and sprouting of new branches, the latter being a feature of lymphangiogenesis that is partially mediated by VEGF-C and VEGF-D [25]. Improved fluid clearance and immune-cell transport from inflamed synovial tissue, mediated by lymphangiogenesis in the setting of functional maturation of these newly formed lymphatic vessels, contribute to resolution of inflammation [26].

A key pathway in compensatory lymphangiogenesis is the stimulation of LECs by pro-inflammatory cytokines [27]. For example, IL-33 promotes lymphangiogenesis by stimulating the ST2 receptor on LECs, resulting in phosphorylation of Akt and endothelial nitric oxide synthase (eNOS) and production of nitric oxide (NO) [28]. This NO-dependent signaling pathway enhances LEC proliferation, migration and tube formation, all of which are essential for lymphatic vessel growth [28]. IL-33 enhances lymphatic vessel density and improves lymphatic drainage in mouse models of inflammation-induced lymphangiogenesis, suggesting a potential protective role at the early stages of RA [28].

Another important role in compensatory lymphangiogenesis is played by the interaction of LECs with immune cells [29]. For example, dendritic cells (DCs) and T cells interact within afferent lymphatic capillaries and can modulate lymphatic function [30]. Using intravital microscopy, Hunter et al. observed that DCs and antigen-experienced T cells were frequently arrested or clustered within lymphatic capillaries. Such prolonged interactions may impact on immune-cell transport and local remodeling of the lymphatic endothelium [30]. M2-like macrophages may also support lymphangiogenesis by releasing VEGF-C and other factors that stimulate lymphatic endothelial cells [31]. In a mouse model of osteoarthritis, exosomes derived from M2-like macrophages increased LEC proliferation and lymphatic vessel growth [31]. Although this finding identifies a possible macrophage–LEC signaling mechanism, its relevance to RA remains to be established.

But an increase in lymphatic vessel growth does not necessarily improve lymphatic function. When vessel growth exceeds the capacity for proper maturation, newly formed lymphatic vessels may remain permeable, poorly organized, or unable to transport interstitial fluid efficiently [32,33]. This distinction between vessel growth and vessel function has also been observed in a mouse model of obesity-associated lymphatic dysfunction, in which lymphangiogenesis coincided with increased vessel permeability and reduced lymphatic drainage [34]. A comparable process may occur in RA, where persistent inflammatory signaling can disrupt lymphatic vessel maturation and reduce drainage from the synovium [35,36]. The resulting fluid and immune-cell retention may then sustain synovial inflammation. The long-term effect of compensatory lymphangiogenesis therefore depends not only on signals that control vessel growth but also on whether newly formed vessels mature, maintain endothelial integrity, and restore drainage within the inflamed synovium.

### 2.2. Lymphatic Drainage Failure During Disease Progression

As RA advances, compensatory lymphangiogenesis may no longer preserve lymphatic function, and the lymphatic network can progress from adaptive remodeling to drainage failure and structural regression. This later stage may involve a reduction in lymphatic vessel density, weaker contractions of collecting lymphatic vessels, and increased vessel permeability. Together, these defects can promote the retention of interstitial fluid and inflammatory mediators within the synovium [34]. Loss of lymphatic drainage can sustain synovial inflammation and contribute to joint damage because immune cells, cytokines, and tissue fluid are not efficiently cleared from the affected joint [37].

Prolonged exposure of LECs to inflammatory cytokines and infiltrating immune cells may drive this functional deterioration [38]. TNF-α and IL-1β can suppress the expression of PROX1 and VEGFR3 in LECs, weakening lymphatic endothelial identity and favoring vessel regression. In a mouse model of nonalcoholic steatohepatitis (NASH), oxidized low-density lipoprotein (oxLDL) reduced PROX1 expression in LECs, which in turn decreased lymphatic-specific gene expression, impaired LEC metabolism, and reorganized cell–cell junctions—paradoxically tightening the normally highly permeable lymphatic capillary junctions and thereby impeding protein and fluid drainage rather than causing frank barrier leakage [39]. A comparable pathway may operate in RA, where persistent inflammatory signaling could suppress PROX1 and other genes that maintain lymphatic endothelial identity [40]. Direct evidence for this mechanism in synovial LECs, however, remains limited.

NET accumulation may provide another source of injury to the lymphatic endothelium [41]. NETs contain extracellular histones, MPO, neutrophil elastase, and other granule-derived molecules that can injure endothelial cells. Histones and NET-associated proteases may also destabilize LEC junctions and weaken the lymphatic endothelial barrier. These observations raise the possibility that NET-mediated LEC injury also reduces lymphatic drainage in RA, but this mechanism requires direct validation in synovial lymphatic vessels. NET-associated HMGB1 can activate RAGE signaling in other cell types, but whether an equivalent pathway operates in synovial LECs remains unknown [42].

Changes in the synovial extracellular matrix (ECM) may impose an additional constraint on lymphatic vessel function. In RA, fibroblast-like synoviocytes (FLS) abundantly express fibroblast activation protein-alpha (FAP-alpha), which contributes to their pathogenic activation, migration, invasion, and matrix remodeling [43]. Thus, the fibrotic and inflammatory synovial microenvironment may adversely affect lymphatic vessel function and fluid or immune-cell drainage; however, direct evidence that synovial fibrosis mechanically compresses lymphatic vessels remains limited.

### 2.3. Interactions Between Lymphatic Vessels and Immune Cells in the Synovial Microenvironment

In RA, the synovial microenvironment comprises immune cells, stromal cells, and extracellular matrix components that interact with lymphatic endothelial cells, thereby influencing local inflammation, immune-cell transport, and tissue remodeling [44]. Lymphatic vessels within the synovium are essential for transporting immune cells and cytokines to lymph nodes (LNs), where adaptive immune responses are initiated [35]. In RA, chronic inflammation and joint damage result from aberrant interactions between lymphatic vessels and immune cells.

The interaction between T cells and LECs is critically important. LECs express major histocompatibility complex (MHC) molecules and are capable of presenting antigens to T cells, thereby regulating T-cell activation and tolerance [45]. Humbert et al. demonstrated that LECs express peripheral tissue-restricted antigens (PTAs) and present them to CD8+ T cells, resulting in T-cell deletion and tolerance [45]. In RA, this tolerance mechanism is often defective, leading to the activation of autoreactive T cells. Furthermore, LECs secrete chemokines such as CCL21, which direct T cells toward lymphatic vessels and facilitate their movement to lymph nodes [45]. Dysregulation of chemokine secretion by LECs in RA may result in the accumulation of autoreactive T cells within the synovium.

Another interesting interaction is between LECs and macrophages. Macrophages are abundant in the synovium of patients with RA and play a pivotal role in inflammation and tissue destruction [46,47]. These macrophages produce tumor necrosis factor-alpha (TNF-α) and interleukin-6 (IL-6), which reinforce local inflammation. Macrophages also affect lymphatic remodeling via paracrine signaling to LECs. VEGF-C released by M2-like macrophages activates VEGFR3 on LECs and supports lymphatic vessel growth [31]. In RA, macrophage populations are frequently skewed toward inflammatory states, increasing cytokine production and diminishing support for the formation and maturation of functional lymphatic vessels [48].

DCs link antigen sampling in the synovium with transport of immune cells via afferent lymphatic vessels. After antigen capture in the synovium, DCs migrate to draining lymph nodes (LNs) via afferent lymphatics and present antigens to T cells [49,50,51,52]. DC and T cell interactions within afferent lymphatic capillaries have been observed using intravital microscopy and may alter DC migration towards draining LNs [30]. Some activated DC populations produce vascular endothelial growth factor A (VEGF-A) and VEGF-C that stimulate LEC proliferation and lymphatic vessel sprouting [53]. Dysregulated activation and antigen presentation by DCs may perpetuate autoreactive T cell responses and sustain synovial inflammation in RA [54].

B cells provide another layer of communication between the synovial immune cells and the lymphatic endothelium. B cells accumulate in the inflamed synovium of many RA patients and produce rheumatoid factor (RF) and anti-citrullinated protein antibodies (ACPAs). These autoantibodies form immune complexes that stimulate inflammatory pathways and promote joint damage [55]. B cells can also modulate LEC behavior and the arrangement of immune-cell trafficking to draining lymph nodes. In RA, persistent B-cell activation sustains autoantibody production and may modify interactions between immune cells and lymphatic vessels. However, direct evidence of how B cells impair lymphatic drainage in RA is limited [56].

### 2.4. Limitations in Current Research on Lymphatic Dysfunction in Rheumatoid Arthritis

Although advances have been made to understand the role of the lymphatics in RA, methodological and conceptual gaps still limit the interpretation of current findings. The primary methodological limitation is the absence of standardized in vivo methods for the assessment of lymphatic function. Lymphoscintigraphy is widely used in many clinical investigations and provides qualitative or semi-quantitative data but often fails to detect rapid changes in lymphatic transport or localized vessel changes [57]. Lymphoscintigraphy did not reliably detect early lymphatic abnormalities in patients with chronic venous insufficiency [58]. Although this group of patients is different from RA patients, these findings demonstrate the difficulty of identifying subtle lymphatic abnormalities with standard imaging methods.

Another disadvantage is the reliance on animal models, as none of the experimental systems completely mimic the clinical and immunological course of human RA. Models such as collagen-induced arthritis (CIA) and collagen antibody-induced arthritis (CAIA) induce lymphatic responses that differ from those observed in RA patients, particularly with respect to the extent and timing of lymphangiogenesis and drainage failure [59]. Furthermore, research has mainly focused on synovial lymphatic vessels, while relatively little has been done on draining lymph nodes (LNs), spleen and the interconnections between these compartments [6]. Therefore, the interaction between alterations in joint lymphatics and immune responses within draining lymphoid tissues and peripheral tissues may not be captured in current studies.

Another challenge is patient heterogeneity, as lymphatic abnormalities may differ between clinical and immunological subgroups of RA. However, many studies do not stratify participants by disease stage, autoantibody status or prior treatment, though these may be involved in lymphatic function and treatment outcomes [1]. Prior to the appearance of clinically overt RA, Hähnlein et al. described functional defects in lymph node stromal cells (LNSCs) from seropositive individuals [6]. These data are consistent with early changes in the lymph node stromal compartment that may precede the development of joint symptoms, but do not provide evidence for global lymphatic dysfunction at this early stage. The timing and sequence of the initial lymphatic changes are not clear as most available studies focus on established RA.

There are no longitudinal studies of lymphatic function during disease progression in RA. Most of the available data are cross-sectional, however, and it is unclear whether lymphatic dysfunction is a cause or consequence of persistent synovial inflammation [3]. Consequently, current evidence cannot determine if impaired drainage is a cause or effect of inflammation, or a component of a reciprocal process. Further prospective studies with serial imaging, functional assessment of drainage and molecular profiling are required to define these temporal relationships and identify lymphatic biomarkers of disease severity or therapeutic response.

## 3. PROX1-Dependent Regulation of Lymphatic Endothelial Identity in Rheumatoid Arthritis

The maintenance of a functional lymphatic network requires LECs to preserve the transcriptional program that distinguishes them from blood vascular endothelial cells. PROX1 occupies a central position in this program by regulating genes involved in lymphatic identity, vessel growth, junctional organization, valve maintenance, and immune-cell trafficking. Inflammatory and metabolic stress may disrupt this regulatory network by altering PROX1 expression or transcriptional activity, although direct evidence from synovial LECs in RA remains limited. This section therefore begins with the established functions of PROX1 before examining how disease-associated signals may destabilize the PROX1-dependent lymphatic phenotype.

### 3.1. PROX1 as a Core Regulator of Lymphatic Endothelial Identity and Function

PROX1 is a homeobox transcription factor that directs the development of lymphatic endothelial cells (LECs) and helps preserve their identity after development [60]. During embryogenesis, PROX1 expression in venous endothelial progenitors initiates the transcriptional program that specifies lymphatic endothelial identity [60]. Adult LECs continue to express PROX1, which maintains the gene-expression program required for the lymphatic endothelial phenotype, vessel integrity, immune-cell transport, and interstitial fluid drainage [60].

PROX1 preserves lymphatic endothelial identity by regulating a network of genes that distinguish LECs from blood vascular endothelial cells [61]. Among its downstream targets, VEGFR3 enables LECs to respond to the lymphangiogenic ligands VEGF-C and VEGF-D and thereby supports lymphatic vessel growth and remodeling [62]. PROX1 also regulates genes associated with lymphatic endothelial specialization, including LYVE1, PDPN, and CCL21 [60]. Mishima et al. demonstrated that PROX1 upregulates expression of the integrin α9 in LECs, thus connecting the PROX1 transcriptional program to lymphatic endothelial adhesion, migration, and vessel morphogenesis [62]. Together, these transcriptional targets reveal mechanisms by which sustained PROX1 expression maintains lymphatic endothelial identity.

PROX1 is essential for the development and maintenance of lymphatic valves, which prevent retrograde flow and facilitate unidirectional lymph transport [63]. Cha et al. reported that YAP and TAZ supported PROX1 expression in valve-forming LECs downstream of VEGF-C signaling. LEC-specific deletion of YAP and TAZ reduced PROX1 expression and was accompanied by progressive valve degeneration [63]. These results connect YAP/TAZ-dependent signaling with the transcriptional program that preserves valve structure, although they do not establish that PROX1 loss alone accounts for the entire phenotype. In RA, persistent inflammatory signaling may suppress PROX1-dependent programs in LECs and thereby weaken vessel identity, barrier integrity, or valve maintenance. Whether this pathway directly causes valve failure and lymphatic stasis in RA, however, remains to be determined.

PROX1 may influence communication between LECs and immune cells by maintaining the transcriptional program that supports lymphatic endothelial specialization [64]. LECs express CCL21, which contributes to the guidance of CCR7-expressing dendritic cells and T cells toward lymphatic vessels and draining lymph nodes. LECs can also express MHC molecules and participate in antigen presentation, thereby modulating T-cell responses [45,65]. In RA, reduced PROX1 activity may disrupt lymphatic endothelial identity, CCL21-dependent immune-cell transport, and communication between LECs and infiltrating leukocytes. Whether these changes directly cause autoreactive T-cell retention in the synovium remains to be determined.

### 3.2. Inflammatory Regulation of PROX1 Expression and Activity

PROX1 expression in LECs can change in response to inflammatory and metabolic stress, but the direction and functional consequences of this regulation depend on the stimulus and disease context [39]. A comparable mechanism may operate in RA, where sustained inflammatory signaling could reduce PROX1-dependent lymphatic programs and weaken vessel identity or drainage. Direct evidence for this pathway in synovial LECs remains limited.

Anti-inflammatory signals may help preserve LEC phenotype by reducing the inflammatory stress that destabilizes lymphatic endothelial programs [9,28]. VEGF-C signaling can help maintain PROX1 expression in specific LEC populations, particularly valve-forming cells in which YAP/TAZ-dependent signaling supports valve development and maintenance [63]. Together, these observations indicate that PROX1-dependent lymphatic programs respond to inflammatory, metabolic, and growth-factor signals, although the direction of regulation differs among experimental settings.

Post-translational modification provides another mechanism that controls PROX1 transcriptional activity. SUMOylation of PROX1 at lysine 556 supports its DNA-binding and transcriptional activity, including its capacity to induce VEGFR3 expression [66]. Pan et al. reported that the SUMOylation-deficient PROX1-K556R mutant showed reduced DNA binding and failed to induce a complete lymphatic endothelial gene program in blood vascular endothelial cells [66]. Whether inflammatory signals directly alter PROX1 SUMOylation or other protein modifications in LECs remains unclear.

Epigenetic mechanisms may also influence PROX1 expression, but their roles in adult LECs under chronic inflammatory conditions remain poorly defined. Studies of blood vascular endothelial cells have shown that atheroprone or disturbed flow can alter DNMT-dependent DNA methylation and promote endothelial-to-mesenchymal transition (EndMT) [67]. Whether inflammatory cytokines induce PROX1 promoter methylation in synovial LECs and thereby destabilize lymphatic endothelial identity in RA remains an untested hypothesis.

### 3.3. LEC Heterogeneity and Disease-Associated Phenotypic Transitions from a Single-Cell Perspective

Recent advances in single-cell RNA sequencing (scRNA-seq) have revealed the heterogeneity of LECs in health and disease [68]. LECs are not a homogeneous population but consist of multiple subpopulations with distinct molecular profiles and functions [69,70]. In RA, the heterogeneity of LECs may contribute to the dynamic changes in lymphatic function during disease progression.

Lymphatic valve endothelial cells (LVECs) are an important subpopulation of LECs and are critical for valve formation and function [63]. LVECs express high levels of PROX1, FOXC2 and GATA2, which are required for valve development [63]. In RA, dysregulation of LVECs may cause loss of valve function resulting in lymphatic stasis. Another important subpopulation is that of capillary LECs, which are responsible for immune-cell trafficking and fluid uptake [68]. Capillary LECs are characterized by high levels of LYVE-1 and podoplanin expression that are required for their function [68]. RA capillary LEC dysfunction may impair fluid drainage and immune-cell trafficking.

The heterogeneity of LECs is modulated by the surrounding microenvironment [71]. For example, exposure of LECs to pro-inflammatory cytokines induces differentiation of LECs into a pro-inflammatory subpopulation, whereas anti-inflammatory cytokines induce differentiation into a reparative subpopulation [68]. This dynamic differentiation is essential for the lymphatic system to adapt to chronic inflammation. An imbalance between pro-inflammatory and reparative LEC subpopulations may drive lymphatic dysfunction and disease progression in RA.

## 4. Context-Dependent Regulation of Lymphatic Function by IL-33/ST2 Signaling

IL-33 regulates lymphatic endothelial cell behavior depending on the molecular form of IL-33, the cellular origin, the local concentration and inflammatory environment. IL-33/ST2 signaling promotes proliferation, migration and lymphangiogenesis of LECs through Akt/eNOS-dependent nitric oxide (NO) production. Chronic inflammation may alter this response, implicating excessive or dysregulated NO signaling in impaired lymphatic contraction and drainage. In order to clarify this context-dependent activity, it is necessary to clarify the biologically active forms of IL-33 and to identify the cells responsible for their production or release.

### 4.1. Biologically Active Forms and Cellular Sources of IL-33

IL-33 is a multifunctional cytokine that exists in two major biological forms, full-length IL-33 (FLIL33) and mature IL-33 (MIL33) [72]. Under physiological conditions, FLIL33 is a nuclear protein localized in the nuclei of endothelial, epithelial, and fibroblast-like cells [72]. FLIL33 is released into the extracellular space in response to cell damage or activation and is processed by proteases such as neutrophil elastase and cathepsin G to yield MIL33 [73]. Neutrophil elastase and cathepsin G can cleave full-length IL-33 into more active mature forms. Extracellular oxidation can rapidly inhibit IL-33 activity by interfering with its ability to engage ST2 [73].

IL-33 is produced by a variety of cell types including endothelial cells, epithelial cells, fibroblasts and immune cells [74,75]. IL-33 is increased in synovial fluid compared to serum and correlates with disease activity in RA patients. Recent single-cell and tissue studies have indicated that IL-33/ST2 signaling drives the differentiation of monocytes into pro-inflammatory macrophages in the synovial microenvironment, thus amplifying synovial inflammation [76].

IL-33 production is regulated by a variety of factors, including pro-inflammatory cytokines, oxidative stress, and mechanical stress [77]. For example, TNF-α and IL-1β can induce IL-33 expression in endothelial cells and FLSs [13,78]. Additionally, oxidative stress can trigger the release of IL-33 from cells by inducing cell damage [72]. Trichloroethylene (TCE) exposure was found to alter the gut microbiome and IL-33 production, suggesting a role for environmental factors in IL-33 regulation [79].

### 4.2. Context-Dependent Regulation of Lymphatic NO Signaling by IL-33/ST2

The ST2 receptor is a member of the IL-1 receptor family and is the primary receptor for IL-33 [72]. ST2 exists in two main isoforms: a membrane-bound form (ST2L) and a soluble form (sST2) [80]. ST2L is expressed on immune cells such as T cells, B cells, macrophages, and ILC2s, while sST2 is a decoy receptor that binds to IL-33 and inhibits its signaling [81,82]. The binding of IL-33 to ST2L initiates a signaling cascade that involves the recruitment of the adaptor protein MyD88, leading to the activation of NF-κB and MAPK pathways [28].

The IL-33/ST2 axis regulates lymphatic function through dual modulation of NO production, which is one of the main mechanisms [28]. This NO-dependent pathway is essential for the pro-lymphangiogenic effects of IL-33 in early phases of RA.

But too much NO can impair lymphatic function. Increased NO levels correlated with reduced lymphatic contractility and drainage [34]. Also, NO can react with superoxide to form peroxynitrite, a reactive nitrogen species that damages LECs and impairs lymphatic function [83]. The IL-33/ST2 axis has a dual role in the regulation of NO production, showing beneficial effects at low concentrations and detrimental effects at high concentrations.

The IL-33/ST2 axis microenvironment further modulates the dual regulation of NO. For example, IL-33 is able to induce excessive NO production in the presence of pro-inflammatory cytokines such as TNF-α, leading to lymphatic dysfunction [28]. On the other hand, IL-33 promotes physiological NO production in the presence of anti-inflammatory cytokines such as IL-10, and ultimately enhances lymphatic function [9]. These results highlight a complex and context-dependent role for the IL-33/ST2 axis in lymphatic regulation.

## 5. Potential Mechanisms of NET-Mediated Lymphatic Endothelial Injury

NETs contain extracellular DNA, histones, myeloperoxidase, neutrophil elastase, cathepsin G, and damage-associated proteins that can alter endothelial viability, junctional integrity, oxidative balance, and extracellular matrix organization. Experimental studies outside RA provide direct evidence that NET accumulation can injure lymphatic endothelium and impair lymphatic drainage. But whether the same components directly damage synovial LECs in RA has not been established. The following sections distinguish the direct effects that individual NET components may exert on lymphatic endothelial integrity from receptor-mediated pathways that remain mechanistically plausible but incompletely validated.

### 5.1. Direct Effects of NET Components on Lymphatic Endothelial Integrity

NETs are composed of a variety of components, including decondensed chromatin, histones, MPO, elastase, and cathepsin G, which can directly damage the LEC barrier [16]. In non-RA experimental models, the histones in NETs are particularly damaging, as they can disrupt the endothelial cell membrane and induce cell death [84]. Extracellular histones are cytotoxic to vascular endothelial cells, while intact NET structures can mechanically disturb blood microvascular perfusion. Whether NET-associated histones exert comparable direct effects on synovial LECs in RA remains uncertain [22]. Nevertheless, in the context of RA, this histone-mediated damage may contribute to LEC dysfunction and lymphatic collapse.

Another component of NETs that damages LECs is MPO, which generates reactive oxygen species (ROS) such as hypochlorous acid (HOCl) [85]. MPO can activate the complement system, leading to further endothelial damage [86]. In RA, the accumulation of MPO in the synovial fluid may contribute to LEC damage and lymphatic dysfunction.

Elastase is another key component of NETs that damages LECs. Elastase can degrade extracellular matrix components such as collagen and elastin, leading to the breakdown of the LEC basement membrane [16]. In non-RA experimental settings, elastase was found to cleave cylindromatosis (CYLD), leading to microtubule disruption and endothelial cell retraction [87]. Although this was not demonstrated in RA LECs, this mechanism may contribute to LEC barrier dysfunction in RA. In RA, cathepsin G activity is elevated in synovial fluid and may contribute to local inflammatory cell recruitment and tissue injury [88]. Although direct evidence in lymphatic endothelial cells remains limited, elevated cathepsin G activity in the RA synovium may plausibly exacerbate lymphatic dysfunction.

### 5.2. Potential Mechanisms of NET-Mediated Lymphatic Endothelial Injury in Rheumatoid Arthritis

NETs contain extracellular DNA, histones, proteases, and damage-associated molecular patterns such as high-mobility group box 1 (HMGB1), all of which may influence lymphatic endothelial homeostasis. Direct evidence that NETs can damage LECs was recently obtained in a non-RA model of intraventricular hemorrhage. In this model, NET accumulation caused acute meningeal LEC injury and lymphatic thrombosis, whereas DNase I-mediated NET degradation promoted LEC recovery and improved lymphatic drainage. However, CX3CR1, rather than RAGE or TLR9, was identified as a key mediator of NET-induced LEC injury [89]. Therefore, RAGE and TLR9 should currently be considered potential, rather than established, mediators of NET-induced LEC dysfunction in RA.

Evidence from other cell types nevertheless supports the biological plausibility of HMGB1/RAGE signaling in NET-mediated inflammation. In a non-RA tumor model, Zha et al. demonstrated that NET-associated HMGB1 bound to RAGE and activated NF-κB signaling in glioma cells, thereby increasing IL-8 secretion and promoting further neutrophil recruitment and NET formation [42]. However, this study did not demonstrate that RAGE activation induces TNF-α, IL-6, IL-1β, ROS, or VEGF production in LECs. Moreover, recombinant HMGB1 has been reported to promote human LEC proliferation, migration, and tube formation in vitro [90], in a non-RA context, suggesting that the effects of HMGB1 on lymphatic endothelium are context-dependent and are not necessarily cytotoxic.

TLR9 may represent another mechanism through which extracellular DNA influences lymphatic endothelial biology. In non-RA LECs derived from the skin, lung, and lymph nodes, TLR9 expression has been reported, although their responsiveness to TLR9 agonists varies according to tissue origin and cellular context [91,92]. Therefore, the presence of TLR9 in non-RA LECs does not necessarily indicate that NET-derived DNA uniformly induces a pro-inflammatory response in RA synovial LECs.

In fibroblasts from patients with systemic sclerosis, Fang et al. showed that CpG-mediated TLR9 activation elicited endogenous TGF-β-dependent profibrotic activation [93]. In addition, NETs have recently been shown to activate the TLR9/MyD88/NF-κB pathway in rheumatoid arthritis fibroblast-like synoviocytes, resulting in increased IL-6 production [94]. These findings provide evidence for TLR9-mediated inflammatory and profibrotic responses in stromal cells but do not establish an equivalent mechanism in LECs.

Collectively, NET-derived HMGB1 and extracellular DNA may activate RAGE- and TLR9-dependent pathways in synovial stromal and immune cells, thereby indirectly aggravating inflammation, fibrosis, and lymphatic dysfunction in rheumatoid arthritis. However, direct evidence that NET-associated HMGB1/RAGE or DNA/TLR9 signaling impairs RA-derived LECs remains lacking. Further studies using RAGE or TLR9 inhibition, receptor knockdown, and NET-treated RA-derived LEC models are required to determine whether these receptors regulate LEC viability, junctional integrity, inflammatory cytokine production, extracellular matrix remodeling, and lymphatic transport.

## 6. An Integrated IL-33–NET–Synovial–Lymphatic Amplification Model in Rheumatoid Arthritis

Current evidence supports a positive feedback loop among IL-33, NETs, and fibroblast-like synoviocytes (FLSs) in the rheumatoid arthritis (RA) synovium. However, the extension of this loop to LEC injury and lymphatic stasis remains a mechanistically plausible model rather than a fully demonstrated pathway in RA. IL-33 levels are elevated in RA synovial fluid and correlate with NET abundance. Mechanistically, IL-33 activates neutrophils through ST2-dependent recruitment of MyD88 and TRAF6, followed by ROS production and activation of the MAPK/ERK, p38 MAPK, and NF-κB pathways, thereby promoting NET formation [95]. NETs subsequently activate RA-FLSs through TLR9 and increase their production of IL-33 and CXCL8. CXCL8 recruits additional neutrophils, whereas FLS-derived IL-33 promotes further NET formation, establishing a directly demonstrated IL-33–NET–FLS amplification loop in the RA synovium [95]. Consistent with this mechanism, ST2-blocking antibodies inhibited IL-33-induced NET formation in vitro, whereas TLR9 antagonism reduced NET accumulation, inflammatory cell infiltration, synovial hyperplasia, cartilage destruction, and bone erosion in IL-33-treated collagen antibody-induced arthritis mice [95].

Whether NETs directly damage synovial LECs in RA has not yet been established. Nevertheless, direct evidence from other inflammatory disease models supports this possibility [89]. These findings provide proof of principle that NETs can directly disrupt lymphatic endothelium, although the responsible receptor and pathological context may differ between meningeal and synovial lymphatics. By contrast, studies showing that NETs mechanically disturb flow through microfluidic capillary plexuses examined blood microvascular networks rather than lymphatic vessels and should not be interpreted as direct evidence of LEC injury or lymphatic obstruction.

The effects of IL-33 on lymphatic vessels are also likely to be context-dependent [28]. Thus, IL-33 may initially stimulate a compensatory lymphangiogenic response rather than exclusively causing lymphatic injury. This interpretation is consistent with studies of TNF-transgenic arthritis showing that early inflammation is accompanied by lymphangiogenesis and expansion of joint-draining lymph nodes, which initially help maintain lymphatic transport. During chronic or advanced arthritis, however, TNF-induced iNOS expression in LECs inhibits the contractile function of adjacent lymphatic smooth muscle cells and reduces lymphatic drainage, and draining lymph nodes may subsequently collapse, resulting in impaired clearance of inflammatory cells and mediators [96,97,98]. Impaired clearance could, in turn, prolong exposure of synovial cells to IL-33, NET components, cytokines, and autoantigens. However, direct evidence that lymphatic stasis itself increases IL-33 release or NET formation in RA is currently lacking.

This proposed lymphatic amplification pathway interacts with multiple established synovial inflammatory circuits. NETs contain citrullinated proteins that can be internalized by RA-FLSs through RAGE–TLR9-dependent mechanisms. This process induces a pro-inflammatory and antigen-presenting phenotype in FLSs and facilitates the activation of autoreactive T and B cell responses against citrullinated antigens [99]. RA-associated anti-citrullinated protein antibodies can, in turn, enhance NET formation, creating reciprocal amplification between NETosis and adaptive autoimmunity. NETs have also been reported to stimulate RA-FLSs to produce IL-6 through TLR9/MyD88/NF-κB signaling, thereby promoting T follicular helper cell differentiation and potentially enhancing B-cell-mediated autoantibody responses [94]. In parallel, IL-33 promotes the differentiation of pro-inflammatory macrophages through MAPK/NF-κB signaling, while these macrophages produce additional IL-33 and TNF, forming another synovial amplification circuit [76]. FLS–osteoclast calcium and cytokine signaling further links persistent synovial activation to osteoclast maturation and bone erosion [100].

FLS-derived lymphangiogenic signals should be distinguished from mechanisms that directly cause lymphatic failure. TNF-α induces VEGF-C expression in RA-FLSs, and VEGF-C concentrations are elevated in RA synovial fluid [101]. Although this response may contribute to synovial lymphangiogenesis, available experimental evidence indicates that enhancement of VEGF-C signaling increases local lymphatic drainage and reduces synovial inflammation and joint destruction in TNF-transgenic mice [96]. Therefore, FLS-derived VEGF-C is more appropriately interpreted as part of a compensatory lymphangiogenic response than as a direct cause of lymphatic stasis. Whether chronically generated lymphatic vessels are structurally abnormal or functionally insufficient under sustained inflammation requires further investigation.

B cells may contribute to lymphatic dysfunction through a mechanism distinct from VEGF-C secretion. In TNF-transgenic mice, CD23-positive/CD21-high B cells translocate from lymph node follicles into lymphatic sinus spaces during an arthritic flare. This translocation is associated with draining lymph node collapse and decreased afferent lymphatic flow [97]. B-cell depletion removes the obstructing nodal B-cell population, restores passive lymphatic transport, and ameliorates joint inflammation [98]. These findings provide a more direct mechanistic link between B cells and impaired lymphatic drainage than the currently unsupported proposal that B-cell-derived VEGF-C causes dysfunctional lymphangiogenesis.

**Figure 1:**
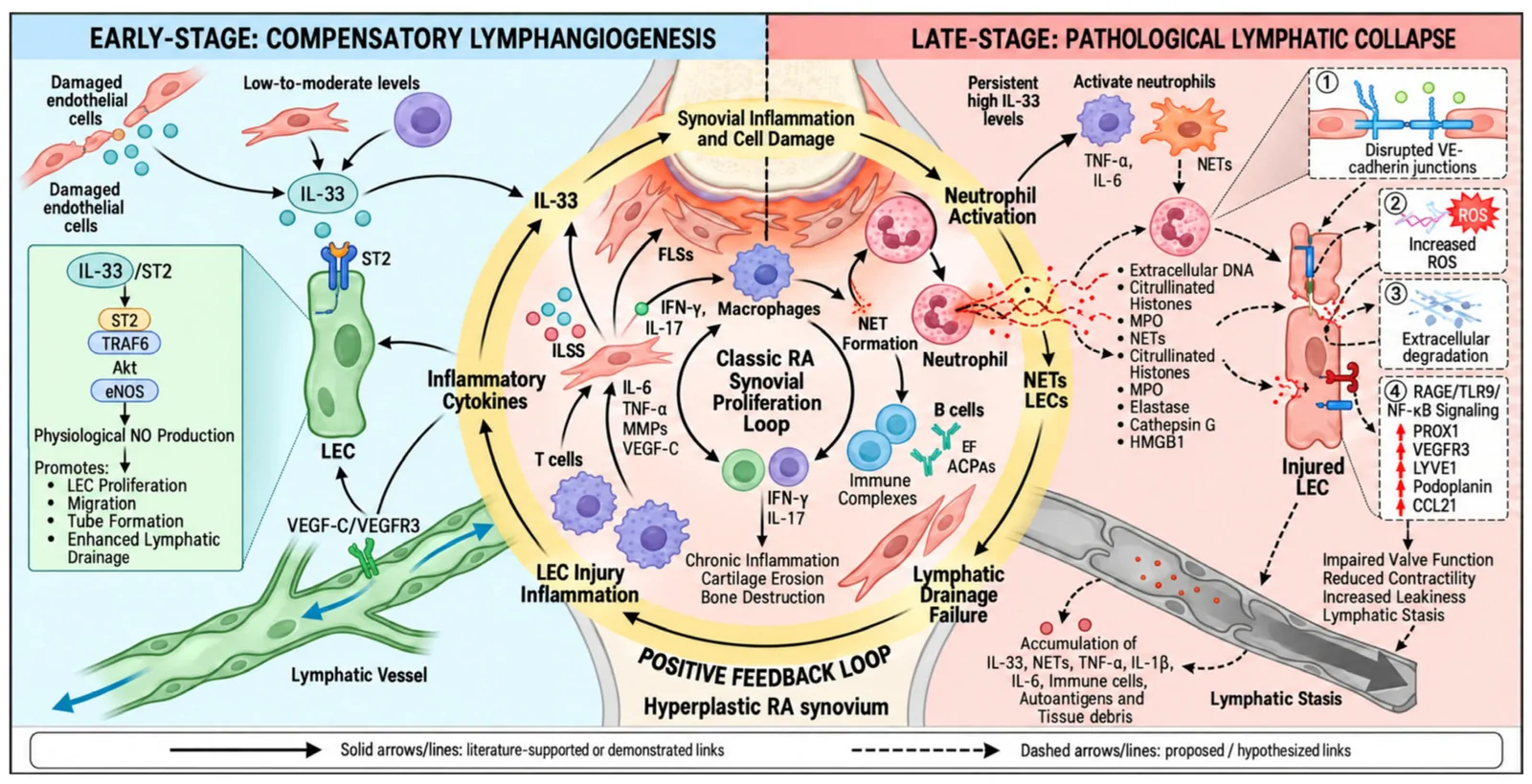
Proposed IL-33–NET–synovial–lymphatic amplification model in rheumatoid arthritis. During early inflammation, IL-33/ST2 and VEGF-C/VEGFR3 signaling promote compensatory lymphangiogenesis. With persistent inflammation, IL-33-driven neutrophil activation and NET formation may injure LECs, weaken the PROX1-dependent lymphatic program, and impair lymphatic drainage. Reduced clearance may then promote the retention of inflammatory mediators and further amplify synovial inflammation and joint damage. Abbreviations: FLS, fibroblast-like synoviocytes; ACPA, anti-citrullinated protein antibodies; RF, rheumatoid factor; MPO, myeloperoxidase; ROS, reactive oxygen species. This figure was created in BioRender (2026).

Taken together, these findings support a stage-dependent integrated model. During early inflammation, IL-33- and VEGF-C-associated lymphangiogenesis may partially compensate for the increased requirement for fluid, antigen, and immune-cell clearance, although IL-33 simultaneously promotes NET formation. As inflammation persists, the IL-33–NET–FLS and IL-33–macrophage feedback circuits increase the local burden of cytokines, NET components, and recruited neutrophils. Concurrent impairment of collecting lymphatic contraction and possible NET-mediated LEC injury may progressively reduce inflammatory clearance, thereby reinforcing synovial inflammation. During advanced disease and arthritic flares, draining lymph node collapse, B-cell occupation of lymphatic sinuses, and severely reduced lymphatic transport may further promote the retention of inflammatory mediators, while FLS–osteoclast interactions drive cartilage and bone destruction. This model integrates established synovial feedback mechanisms with documented lymphatic abnormalities in inflammatory arthritis, but direct longitudinal evidence connecting IL-33, NET-induced synovial LEC injury, and lymphatic stasis across different stages of RA remains to be obtained. The key pathways of this process are summarized in Figure 1.

## 7. Conclusions

In summary, accumulating evidence suggests that lymphatic dysfunction is not only a consequence of rheumatoid arthritis, but also potentially contributes to persistent synovial inflammation and disease progression. The present review highlights the convergence of the IL-33/ST2 axis, NET-mediated lymphatic endothelial injury and PROX1 dysregulation in the formation of a self-amplifying pathogenic loop. These findings give new insights into the biology of RA and suggest promising avenues for the development of biomarkers and targeted therapeutic strategies.

## Data Availability

No new data were created or analyzed in this study. Data sharing is not applicable to this article.
